# Systematic palynology in Korean Piperales with special focus on its exine surface ornamentation and orbicule morphology

**DOI:** 10.1038/s41598-022-08105-3

**Published:** 2022-03-09

**Authors:** Min-Kyeong Oak, Sungyu Yang, Goya Choi, Jun-Ho Song

**Affiliations:** 1 Temperate Middle Part Plant Conservation Team, Sejong National Arboretum, Sejong, 30106 Republic of Korea; 2 Herbal Medicine Resources Research Center, Korea Institute of Oriental Medicine, Naju, 58245 Republic of Korea

**Keywords:** Plant sciences, Natural variation in plants

## Abstract

The pollen and orbicule morphology of the Korean Piperales (*Aristolochia*, *Asarum*, *Houttuynia*, *Piper*, and *Saururus*) were investigated via scanning electron microscopy. Piperales pollen is a monad, its size ranging from very small to large (*P* = 7.78–51.4 μm, *E* = 6.68–43.1 μm), and having a mainly circular to sub-circular shape. The aperture type is constant in the genus [inaperturate (*Aristolochia*), tri to pentaporate (*Asarum*), and monosulcate (*Houttuynia*, *Piper*, and *Saururus*)]. There are four distinct types of exine ornamentation: Fossulate with perforate, microreticulate with gemmae, microperforate with granula, and microechinate. The orbicules (minute sporopollenin granules) were observed in all studied taxa and thus, may be a possible symplesiomorphic characteristic of Piperales. Further, the observed orbicule surface ornamentation was similar to pollen exine patterns, for example muri, gemmae, or granula. This resemblance between orbicule and pollen exine ornamentation may imply a similar biosynthesis pattern of sporopollenin of pollen exine and orbicules. The phenogram resulting from a cluster analysis using palynological characters was generally consistent with the known molecular phylogeny of Piperales. This initial study will help understand the palynological diversity and provide detailed information of pollen and orbicule characteristics in Piperales.

## Introduction

Palynological traits have been considered as useful diagnostic and systematic characteristics since the work of Erdtman^1^. They often provide essential evidence to recognize genera and/or species, and resolve their phylogenetic relationship. In particular, exine ornamentation features on pollen outer walls support molecular phylogenetic hypothesis and contribute to defining their systematic groups^2,3^. The pollen exine consists of sporopollenin; a tough resistant biopolymer that protects the vulnerable gametes from a wide range of physical and chemical forces^4,5^.

Orbicules^6^ or Ubisch bodies^7^, minute granules on inner locule walls of mature anthers, are also composed of sporopollenin similar to the pollen exine^8–10^. The pro-orbicules (as lipid droplets) or orbicule precursors are coated with sporopollenin synchronously with the growing pollen exine^11^. Thus, the orbicule surface pattern often resembles that of the pollen exine ornamentation^9,12–14^. Orbicules are generally spherically-shaped and smooth; however various shapes such as granulate or microperforate, and even echinate, can be found depending on the species^15^. The distribution pattern in flowering plants has recently been reviewed^16,17^. Studies have demonstrated that orbicules are usually common in the ANA grade, magnoliids, and monocots, but are absent in late-branching clades in angiosperms^16,17^. More recently, comprehensive palynological studies have aimed to verify pollen morphology and orbicule diversity^2,3,14,18^.

Piperales Bercht. & J. Presl. is one of the orders in the magnoliid clade and consists of three families; Aristolochiaceae Juss. (Dutchman’s pipe family), Piperaceae Giseke (black pepper family), and Saururaceae Martynov. (lizard’s-tail family)^19^. This order comprises approximately 4300 species and is the most diverse within the magnoliids^20^.

Some publications about the general pollen morphology^21–29^ and orbicule/tapetum characteristics^30–33^ in selected groups of the Piperales have provided good descriptions of the pollen and orbicule/tapetum; however, to date, no studies have investigated the relationship between pollen and orbicule characteristics.

Korean Piperales includes the three generally accepted families and 9–13 species representing five genera (*Aristolochia* L., *Asarum* L., *Houttuynia* Thunb., *Piper* L., and *Saururus* L.). Various taxonomic studies in the selected group of Korean Piperales have been conducted, including taxonomic review^34,35^, phylogeny^36^, morphology^37^, and leaf micromorphology^38^. However, a comprehensive palynological study has not been conducted using scanning electron microscopy (SEM).

Our study aims to (1) illustrate and document the pollen and orbicule of Korean Piperales in detail, (2) discuss the variation in pollen and orbicule characteristics and evaluate their potential systematic value, and (3) infer the relationship between pollen and orbicule surface ornamentation pattern in Piperales, for the first time.

## Results

**Figure 1:**
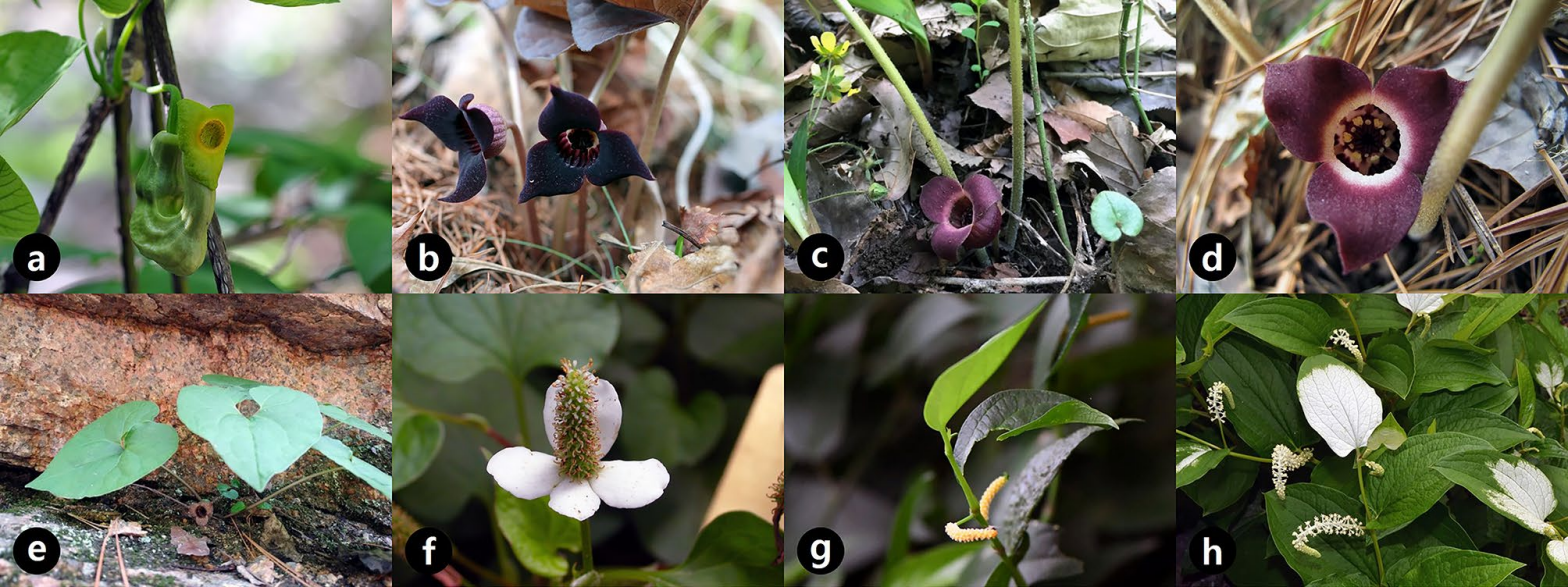
Flowers and inflorescence of representative genera of the Korean Piperales (**a**) *Aristolochia manshuriensis*. (**b**) *Asarum koreanum*. (**c**) *Asarum mandshuricum* var. *seoulense*. (**d**) *Asarum sieboldii*. (**e**) *Asarum versicolor*. (**f**) *Houttuynia cordata*. (**g**) *Piper kadsura*. (**h**) *Saururus chinensis*. (**a**–**d**, **f**, **h**) were obtained by Jun-Ho Song, and (**e**, **g**) were obtained by Sungyu Yang.

**Figure 2:**
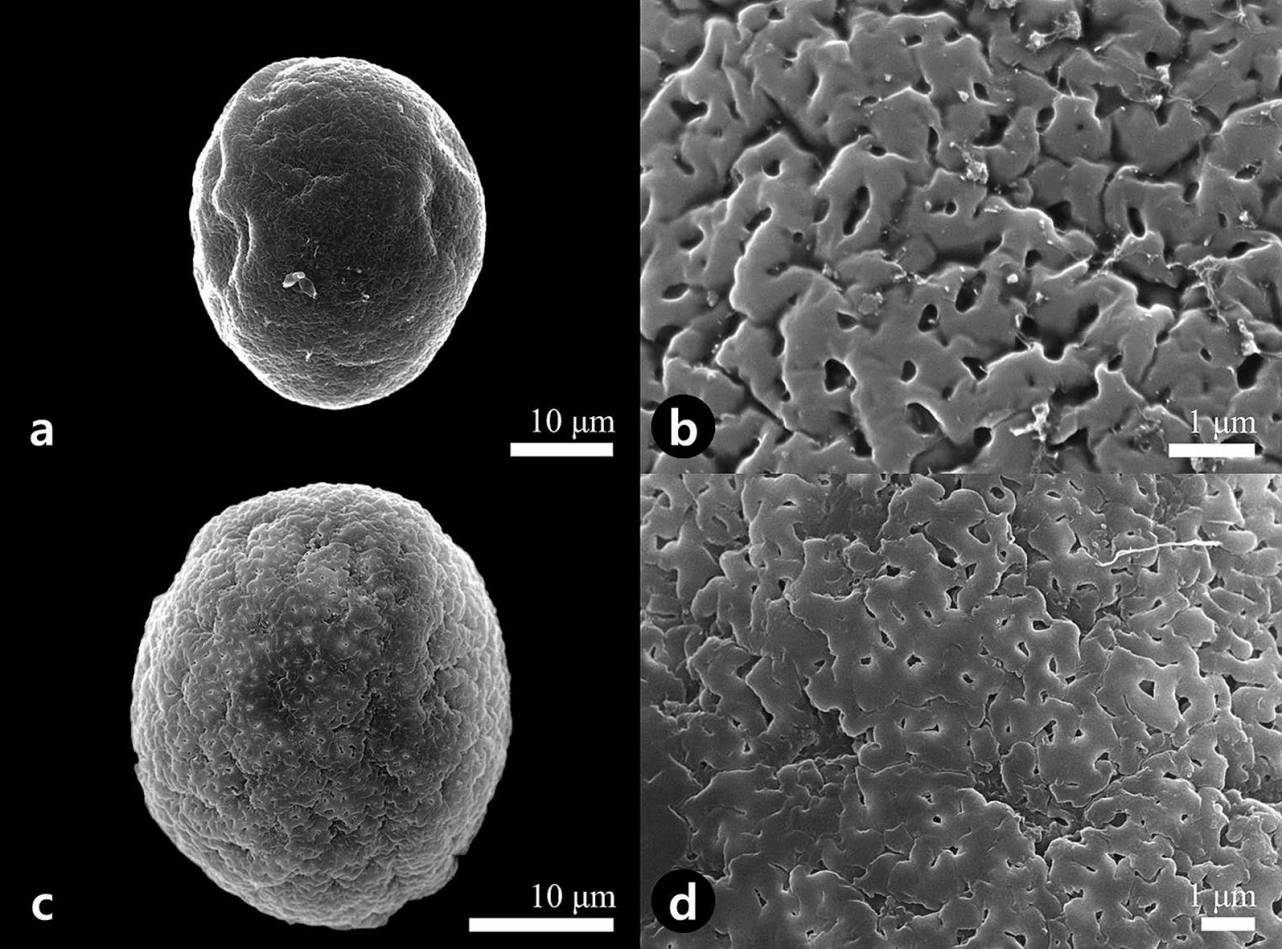
Scanning electron micrographs showing the pollen grains (**a**, **c**) and detailed surface ornamentation (**b**, **d**). (**a**, **b**) *Aristolochia contorta*. (**c**, **d**) *Aristolochia manshuriensis*.

**Figure 3:**
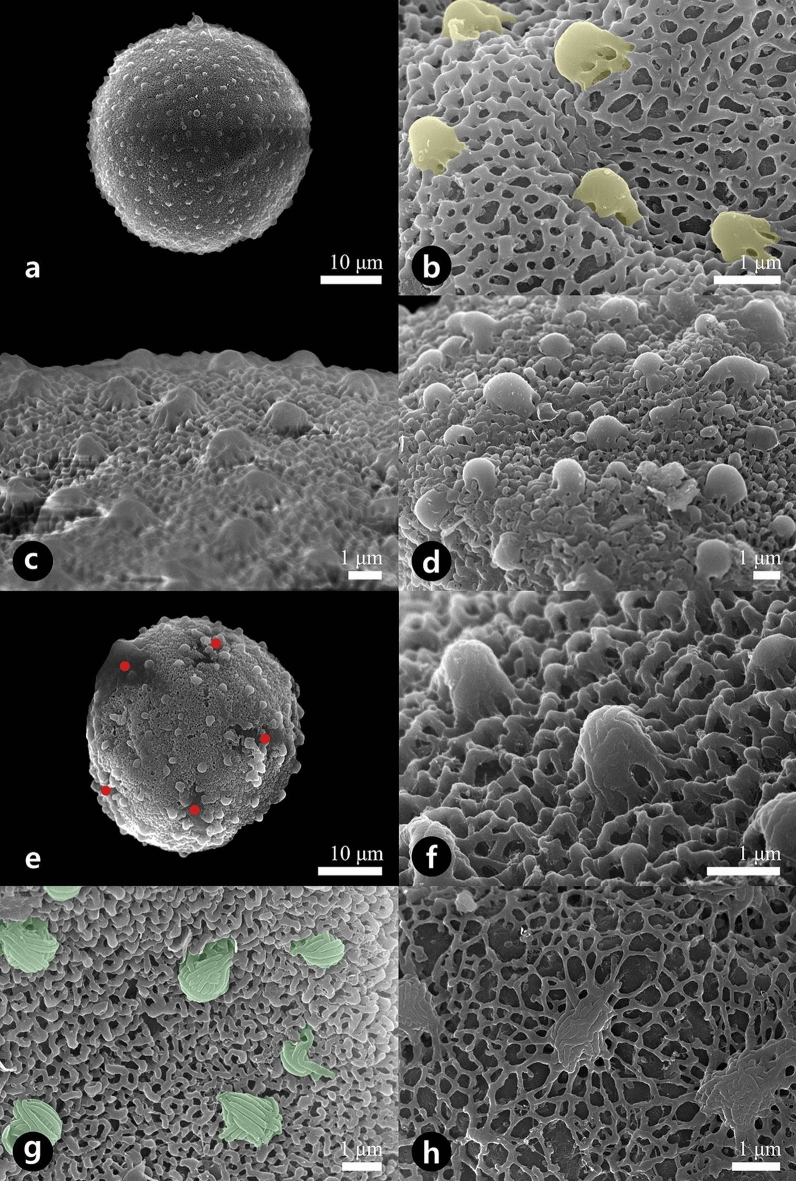
Scanning electron micrographs showing the pollen grains (**a**, **e**) and detailed surface ornamentation (**b**–**d**, **f**–**h**). (**a**, **b**) *Asarum mandshuricum* var. *mandshuricum*. (**c**) *Asarum misandrum*. (**d**) *Asarum versicolor*. (**e**) *Asarum patens*. (**f**) *Asarum mandshuricum* var. *seoulense*. (**g**) *Asarum koreanum*. (**h**) *Asarum sieboldii*. Yellow color indicates smooth-surfaced gemmae and these patterns are correlated with those of Fig. 5e. Green color indicates striate-surfaced gemmae and these patterns are correlated with those of Fig. 5d. Red dots indicate apertures.

**Figure 4:**
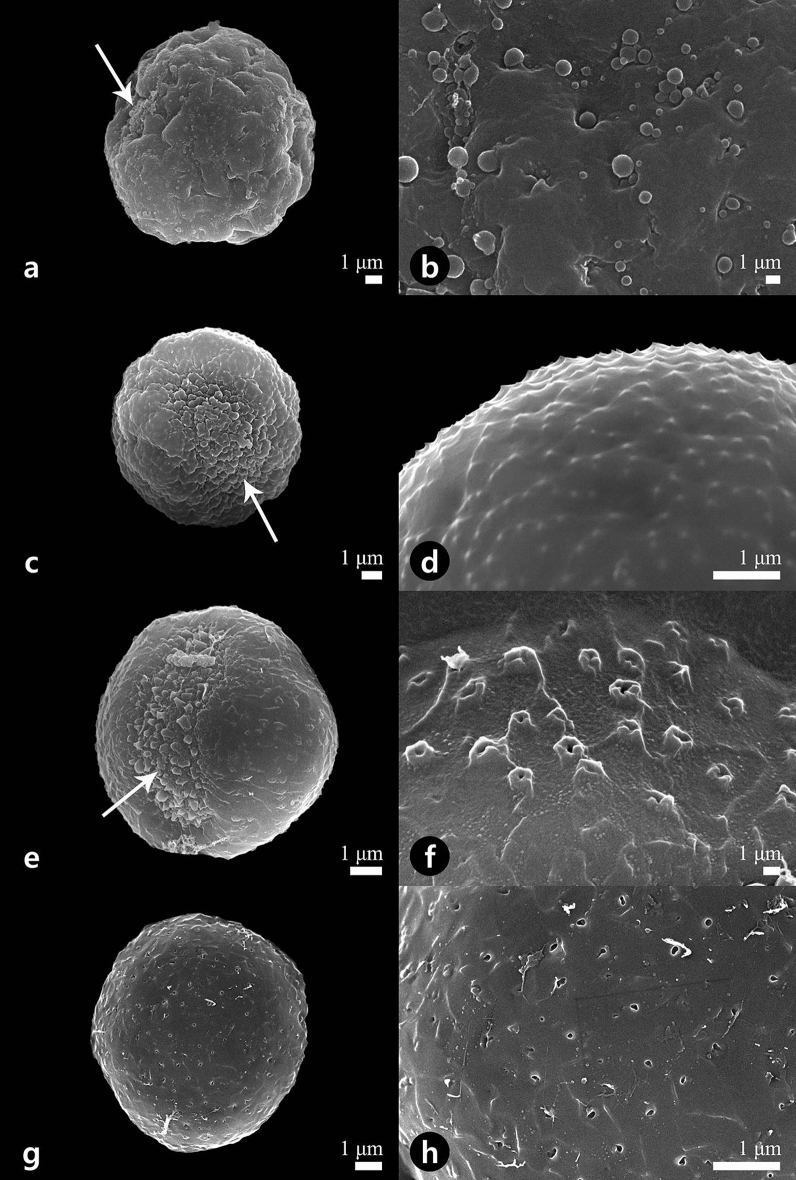
Scanning electron micrographs showing the pollen grains (**a**, **c**, **e**, **g**) and detailed surface ornamentation (**b**, **d**, **f**, **h**). (**a**, **b**) *Houttuynia cordata*. (**c**, **d**) *Piper kadsura*. (**e**–**h**) *Saururus chinensis*. White arrows indicate apertures.

**Figure 5:**
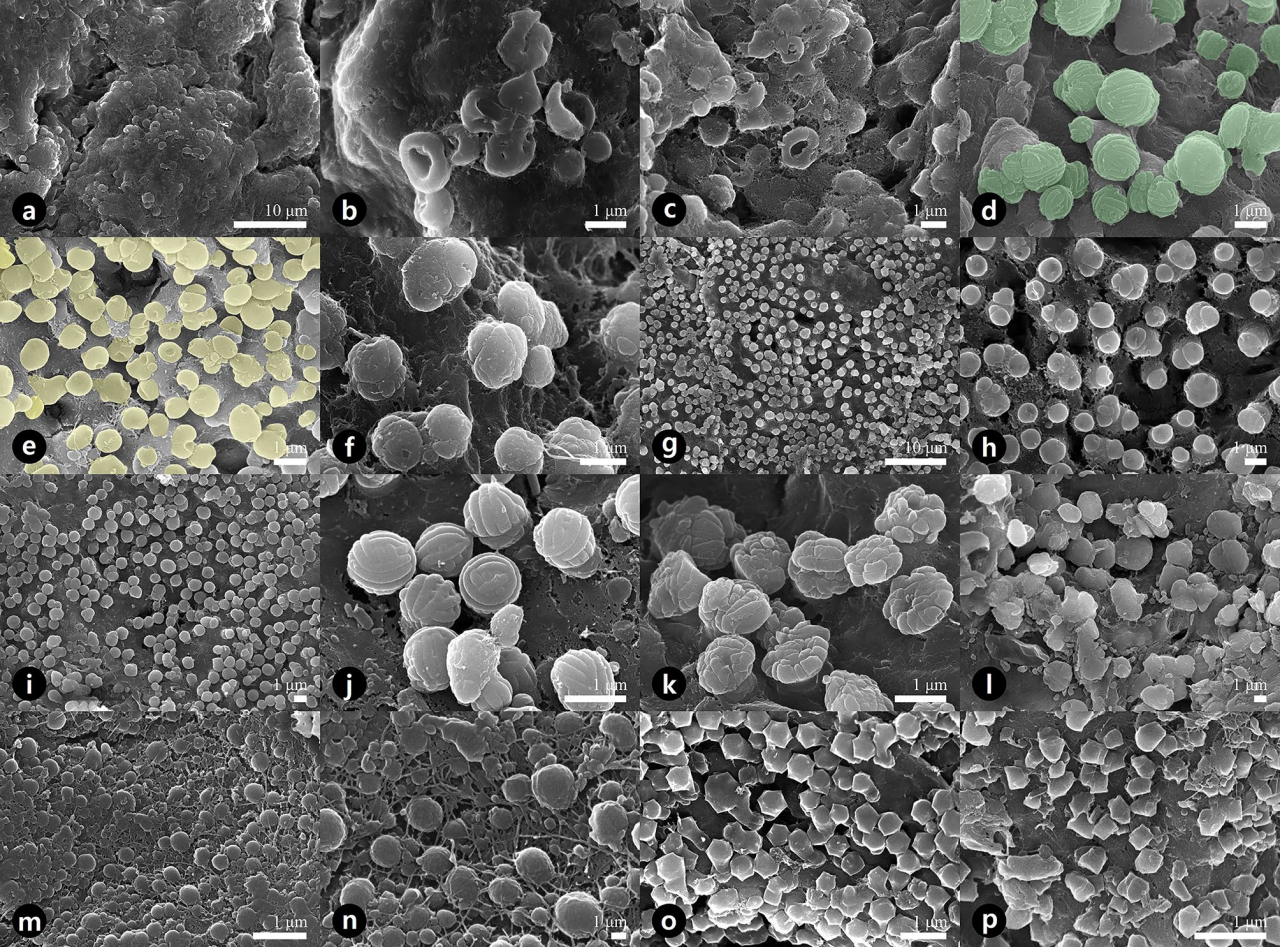
Scanning electron micrographs showing the orbicules on the inner locule walls. (**a**, **b**) *Aristolochia contorta*. (**c**) *Aristolochia manshuriensis*. (**d**) *Asarum koreanum*. (**e**) *Asarum mandshuricum* var. *mandshuricum*. (**f**) *Asarum mandshuricum* var. *seoulense*. (**g**, **h**) *Asarum misandrum*. (**i**, **j)** *Asarum patens*. (**k**) *Asarum sieboldii*. **l** *Asarum versicolor*. (**m**, **n**) *Houttuynia cordata*. (**o**) *Piper kadsura*. (**p**) *Saururus chinensis*.

The morphological variation of pollen and orbicules within Korean Piperales taxa (Fig. 1) are described. The representative pollen characteristics of all investigated taxa are summarized in Tables 1 and 2. Representative pollen grains and orbicules of Korean Piperales are illustrated in Figs. 2, 3, 4, and 5.

**Table 1:** Overview of pollen morphological characters in the Korean Piperales taxa.

Taxa	A	Pollen grain		O	Muri		Lumen or perforation		Gemmae or granula	
		Polar axis (μm)	Equatorial diameter (μm)		Width (μm)	L/W ratio (μm)	Diameter (μm)	Area (μm^2^)	S	Diameter (μm)
*Aristolochia contorta* (ArC)	in	34.1 ± 1.00	28.5 ± 0.60	fs, pf	0.29–(0.47)–0.74	0.38–(0.79)–1.15	0.16–(0.27)–0.47	0.01–(0.05)–0.12	–	–
*Aristolochia manshuriensis* (ArM)	in	29.9 ± 1.81	26.5 ± 1.51	fs, pf	0.37–(0.56)–0.80	0.59–(0.96)–1.40	0.13–(0.23)–0.42	0.02–(0.05)–0.13	–	–
*Asarum koreanum* (AsK)	tri, tet	38.2 ± 2.01	35.2 ± 2.11	mr, gm	0.14–(0.18)–0.23	0.28–(0.39)–0.50	0.19–(0.25)–0.31	0.02–(0.03)–0.05	st	0.81–(1.32)–1.76
*Asarum mandshuricum* var. *mandshuricum* (AsH)	tri, tet	37.6 ± 2.31	35.4 ± 2.94	mr, gm	0.10–(0.14)–0.20	0.23–(0.36)–0.46	0.14–(0.30)–0.51	0.01–(0.04)–0.11	sm	0.58–(0.84)–1.13
*Asarum mandshuricum* var. *seoulense* (AsS)	tri, tet	36.9 ± 1.84	35.3 ± 1.95	mr, gm	0.12–(0.20)–0.28	0.33–(0.43)–0.53	0.21–(0.33)–0.51	0.05–(0.05)–0.13	st	0.56–(1.12)–1.39
*Asarum misandrum* (AsM)	tri, tet	45.5 ± 3.57	37.2 ± 2.22	mr, gm	0.15–(0.20)–0.26	0.29–(0.40)–0.54	0.21–(0.30)–0.45	0.02–(0.04)–0.08	sm	0.77–(1.01)–1.36
*Asarum patens* (AsP)	tet, pen	40.1 ± 2.05	36.7 ± 2.00	mr, gm	0.09–(0.14)–0.19	0.26–(0.38)–0.46	0.20–(0.33)–0.50	0.02–(0.06)–0.14	st	0.79–(1.06)–1.32
*Asarum sieboldii* (AsB)	tri, tet	39.2 ± 1.30	37.1 ± 1.22	mr, gm	0.08–(0.11)–0.14	0.35–(0.52)–0.74	0.26–(0.60)–0.94	0.02–(0.06)–0.14	st	0.99–(1.29)–1.64
*Asarum versicolor* (AsV)	tri, tet	38.9 ± 3.40	36.3 ± 3.79	mr, gm	0.14–(0.22)–0.32	0.26–(0.35)–0.46	0.14–(0.23)–0.32	0.01–(0.03)–0.05	sm	1.33–(1.86)–2.65
*Houttuynia cordata* (HC)	mono	14.5 ± 0.29	14.2 ± 0.20	mp, gr	–	–	0.06–(0.09)–0.13	0.002–(0.003)–0.006	sm	0.10–(0.18)–0.30
*Piper kadsura* (PK)	mono	11.3 ± 0.78	10.1 ± 0.84	me	–	–	–	–	me	0.29–(0.44)–0.57
*Saururus chinensis* (SC)	mono	8.65 ± 0.67	7.70 ± 0.68	mp, gr	–	–	0.09–(0.14)–0.23	0.002–(0.008)–0.014	sm	0.14–(0.26)–0.37

Aperture (A): *in* inaperturate, *mono* monosulcate, *pen* pentaporate, *tet* tetraporate, *tri* triporate. Ornamentation (O): *fs* fossulate, *gm* gemmate, *gr* granulate, *pf* perforate, *me* microechinate, *mp* microperforate, *mr* microreticulate. Gemmae or granula surface (S): *me* microechinate, *sm* smooth, *st* striate. Polar axis and equatorial diameter show mean ± S.D. and muri, lumen or perforation show minimum–(mean)–maximum.

**Table 2:** Overview of orbicule characters in the Korean Piperales taxa.

Taxa	Density	Diameter (μm)	Shape	Surface ornamentation	Association	Correlation with
*Aristolochia contorta* (ArC)	sc	0.81–(1.06)–1.40	dt-shaped	ps	em	Muri
*Aristolochia manshuriensis* (ArM)	sc	0.54–(0.99)–1.58	dt-shaped	ps	em	Muri
*Asarum koreanum* (AsK)	vab	0.93–(1.47)–1.98	wt-shaped	st	ag, em	Gemmae
*Asarum mandshuricum* var. *mandshuricum* (AsH)	vab	0.63–(0.86)–1.08	dt-shaped	ps	ag, em	Gemmae
*Asarum mandshuricum* var. *seoulense* (AsS)	vab	0.98–(1.20)–1.50	dt-shaped	ru	ag, em	Gemmae
*Asarum misandrum* (AsM)	vab	0.90–(1.09)–1.57	dt-shaped	ps	ag, em	Gemmae
*Asarum patens* (AsP)	vab	0.87–(1.05)–1.38	wt-shaped	st	ag, em	Gemmae
*Asarum sieboldii* (AsB)	vab	0.87–(1.05)–1.38	wt-shaped	st	ag, em	Gemmae
*Asarum versicolor* (AsV)	ab	1.24–(2.01)–2.79	dt-shaped	ps	ag, em	Gemmae
*Houttuynia cordata* (HC)	vab	1.22–(1.99)–2.79	sph	ps	con	Granula
*Piper kadsura* (PK)	vab	0.43–(0.51)–0.61	pgp	ms	ag, em	Granula
*Saururus chinensis* (SC)	ab	0.24–(0.35)–0.42	pgp	ps	ag, em	Granula

Density: *ab* abundant, *sc* scattered, *vab* very abundant, shape: *dt-shaped* dought-shaped, *pgp* polygonal prism, *sph* spherical, *wt-shaped* walnut-shaped, Surface ornamentation: *ms* microspine, *ps* psilate, *ru* regulate, *st* striate; association: *ag* aggragated, *con* connected via threads, *em* embedded.

### Size and shape

Pollen grains were shed as monads, and their size varied from very small to large in all studied taxa [*P* = 7.78–(31.2)–51.4 μm, *E* = 6.68–(28.4)–43.1 μm; Table 1]. *Saururus chinensis* possessed the smallest pollen grains [*P* = 7.80–(8.65)–9.91 μm, *E* = 6.72–(7.70)–8.62 μm], whereas *Asarum misandrum* had relatively larger pollen grains [*P* = 39.0–(45.5)–51.4 μm, *E* = 35.1–(37.2)–41.9 μm; Table 1]. The equatorial outline was subprolate (Fig. 2a,c) to prolate-spheroidal (Fig. 4e,g).

### Ambs

The amb (outline in the polar view) was mostly circular to sub-circular (Figs. 3a,e, 4a,c).

### Apertures

Two *Aristolochia* species exhibited no aperture (inaperturate) on the surface of pollen grains (Fig. 2a,c; Table 1). The aperture number of the genus *Asarum* varied from three to five. Triporate, tetraporate, and pentaporate pollen grains were observed in *Asarum* taxa. All pollen grains of *Houttuynia*, *Piper*, and *Saururus* were monosulcate (Fig. 4a,c,e; Table 1).

### Exine ornamentation

Four distinct types of exine ornamentation were defined based on the existence of gemmae or granula; perforate or echinate patterns.

Type I: *Fossulate with perforate*—all species of *Aristolochia* (Fig. 2b,d).

All studied taxa within *Aristolochia* had semitectate exines. The surface ornamentation pattern was fossulate with well-developed muri arranged in an irregular shape. The murus width ranged between 0.29 and 0.80 μm, and the range of perforation diameter and their area was 0.16–0.47 μm and 0.01–0.13 μm^2^, respectively (Table 1). There were no gemmae or granula on the surface; observed in *A. contorta* (Fig. 2b) and *A. manshuriensis* (Fig. 2d).

Type II: *Microreticulate with gemmae*—all taxa of *Asarum* (Fig. 3b–d,f–h).

The semitectate exine and microreticulate surfaces were found in all taxa from *Asarum*. The microbrochate (microreticulate) pattern was arranged with well-developed muri and lumina in a regular shape. The murus width ranged between 0.08 and 0.32 μm, and the range of luminal diameter and area was 0.14–0.94 μm and 0.01–0.14 μm^2^, respectively (Table 1). All taxa belonging to this type were homobrochate (homoreticulate); however, only *A. sieboldii* was heterobrochate (heteroreticulate) (different sized reticulum) (Fig. 3h). Sculpture elements, gemmae (0.56–2.65 μm in diameter), were distributed on the surface (Table 1). This ornamentation type was divided into two subtypes based on the surface pattern of the gemmae: Type II-1, *microreticulate with smooth-surfaced gemmae*; observed in *A. mandshuricum* var. *mandshuricum* (Fig. 3b), *A. misandrum* (Fig. 3c), and *A. versicolor* (Fig. 3d); type II-2, *microreticulate with striate-surfaced gemmae*; observed in *A. koreanum* (Fig. 3g), *A. mandshuricum* var. *seoulense* (Fig. 3f), *A. patens* (Fig. 3e), and *A. sieboldii* (Fig. 3h).

Type III: *Microperforate with granula*—*Houttuynia* (Fig. 4b) and *Saururus* (Fig. 4f,h).

The smooth exine ornamentation had some tectal microperforations (0.06–0.23 μm in diameter) and was bordered by one to five granula (0.10–0.37 μm in diameter; Table 1); observed in *H. cordata* (Fig. 4b) and *S. chinensis* (Fig. 4f,h).

Type IV: *Microechinate*—*Piper* (Fig. 4d).

The microechinae were distributed regularly on the pollen grain surface without perforation patterns. The aperture membrane ornamented granula (0.29–0.57 μm in diameter; Table 1); observed in *P. kadsura* (Fig. 4d).

### Orbicule morphology

Orbicules were consistently observed in all studied taxa. Orbicule density was classified as scattered, abundant, or very abundant (Table 2). The diameter of the orbicules was 1.01 ± 0.50 μm on average. *Asarum versicolor* had the largest orbicules (2.01 ± 0.42 μm), whereas *Saururus chinensis* had the smallest ones (0.35 ± 0.68 μm; Table 2). Four types of orbicule shape were observed; donut-shaped (i.e., spherical with a central perforation; Fig. 5a–c,e–h,l), walnut-shaped (i.e., spherical with striation patterns; Fig. 5d,i–k), simple-spherical (i.e., spherical without any patterns; Fig. 5m–n), and polygonal prism (Fig. 5o–p). Their surface ornamentation was defined as psilate (Fig. 5a–c,e,g–h,l–n,p), rugulate (Fig. 5f), striate (Fig. 5d,i–k), or microspine (Fig. 5o). The orbicules appeared to be single/embedded (Fig. 5a–c), aggregated/embedded (Fig. 5d–l) or connected via threads (Fig. 5m–n) in the tapetal membrane (Table 2).

### Statistical and cluster analysis

**Figure 6:**
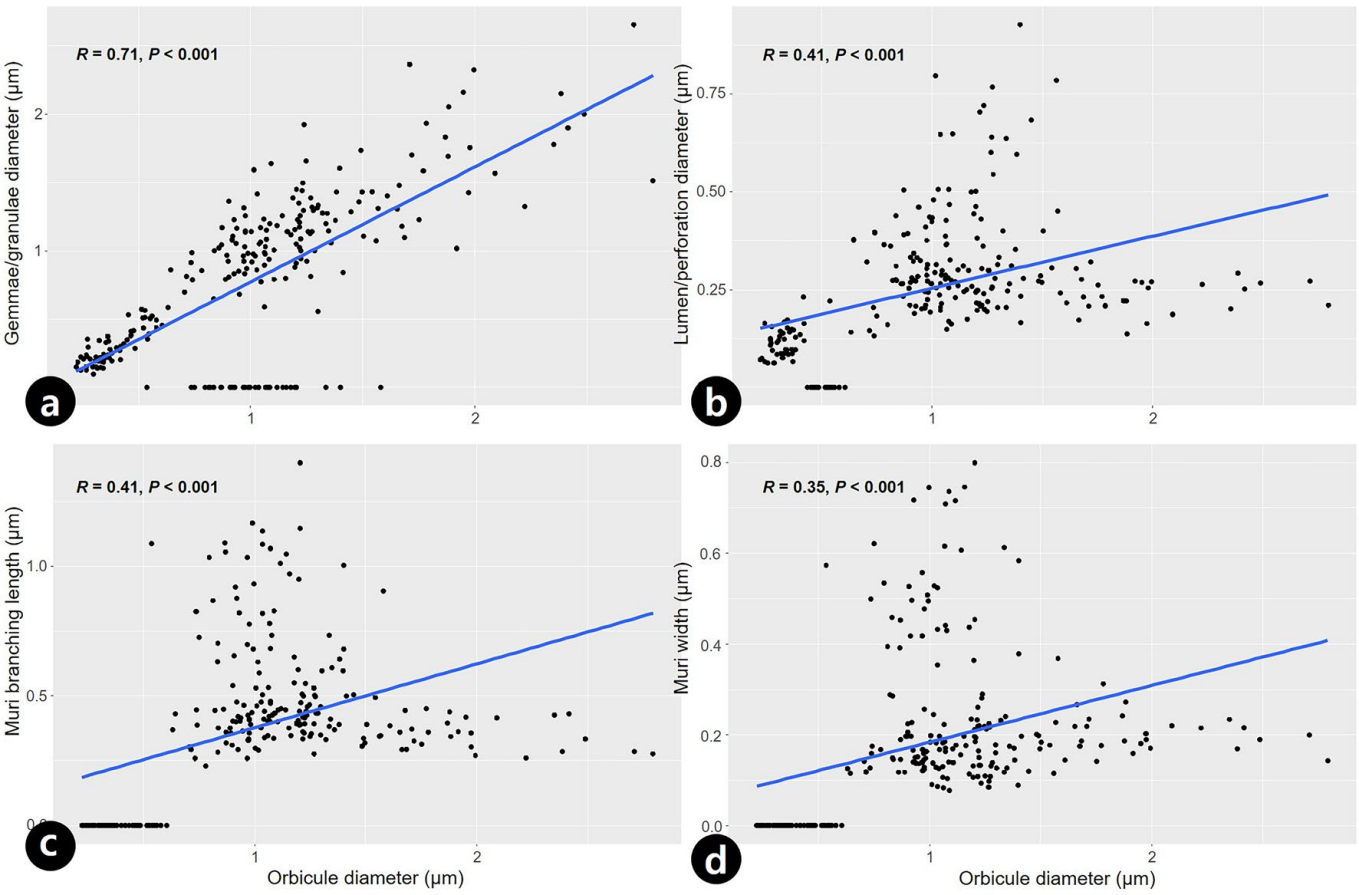
Simple scatter plots of the relationship between orbicule diameter and pollen surface variables (blue line showed regression line) in taxa of Korean Piperales. (**a**) Gemmae/granula diameter (*r* = 0.71, *P* < 0.001). (**b**) Lumen/perforation diameter (*r* = 0.41, *P* < 0.001). (**c**) Muri branching length (*r* = 0.41, *P* < 0.001). (**d**) Muri width (*r* = 0.35, *P* < 0.001) according to orbicule diameter.

**Figure 7:**
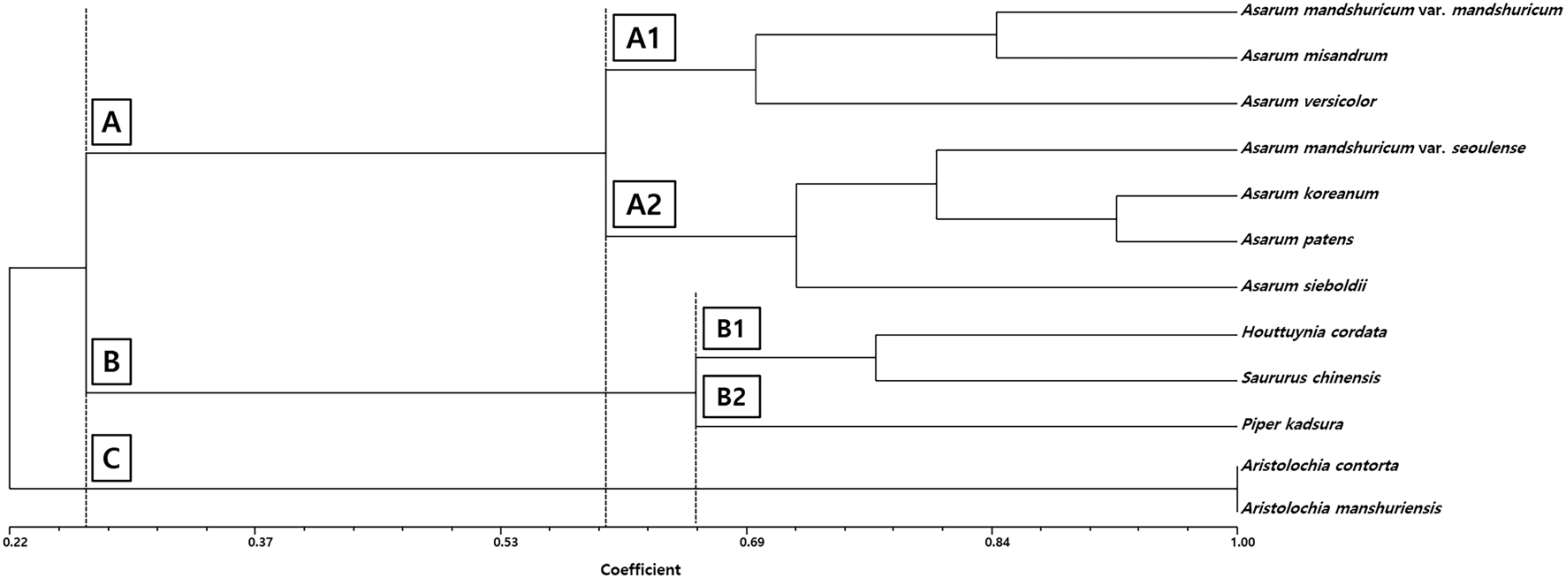
Phenogram for 12 species in the Korean Piperales from cluster analysis based on UPGMA using 13 pollen surface and orbicule palynological characteristics. The vertical dashed line indicates the similarity coefficient.

Orbicule characteristics, such as their diameter, were significantly correlated with the surface pattern of pollen grains. Orbicule diameter was significantly positively correlated with gemmae/granula diameter (*r* = 0.71, *P* < 0.001***; Fig. 6a), lumen/perforation diameter (*r* = 0.41, *P* < 0.001***; Fig. 6b), muri branching length (*r* = 0.41, *P* < 0.001***; Fig. 6c), and muri width (*r* = 0.35, *P* < 0.001***; Fig. 6d). On the unweighted pair group method with arithmetic (UPGMA) phenogram, three major clusters (A, B, and C) were recognized with a similarity coefficient of approximately 0.26 (Fig. 7). The first cluster A comprised two subclusters, A1 and A2. Subcluster A1 contained *Asarum mandshuricum* var. *mandshuricum*, *A. misandrum*, and *A. versicolor*. The adjacent subcluster A2 comprised *Asarum mandshuricum* var. *seoulense*, *A. koreanum*, *A. patens*, and *A. sieboldii*. The second cluster B also comprised two subclusters, B1 and B2. *Houttuynia cordata* and *Saururus chinensis* were clustered in B1. Subcluster B2 contained *Piper kadsura*. The final cluster C, comprised two taxa of *Aristolochia* (Fig. 7).

## Discussion

Using the palynological characteristics identified through SEM analysis, we determined useful key characteristics for identification and a strong correlation between pollen and orbicule surface ornamentation patterns. This was the first study whereby a comprehensive approach using pollen and orbicule morphology covering all genera of Korean Piperales was conducted.

Most previous pollen measurements on the Piperales have been conducted based on acetolysed^21,22,24,29^, air-dried^26^, or fixed/stained pollen grains^28^. In the case of *Aristolochia manshuriensis*, varying pollen size was reported following different preparation techniques and microscopes; stained pollen using stereo microscope [29–(48.46)–65 μm]^28^, acetolyzed pollen using light microscope [41.3–(48.5)–57.3 μm]^29^, critical point dried using SEM [27.4–(29.9)–33.1 μm] (in the present study). Further, the size and shape of pollen grains could be affected by the different preparation methods^2,3,14,39^. The fixation, staining, and acetolysis methods for pollen measuring have side effects such as distortion of shape and change of size. The striking differences in pollen size and distorted pollen shape were due to harmomegathy, a characteristic infolding of pollen grains to accommodate the decrease in cellular volume due to water loss^40,41^. Thus, we recommend the critical point drying preparation to avoid deformation and distortion of pollen grains.

The pollen aperture types of the Korean Piperales were confirmed by comparing previous palynological studies. It is uncertain whether the aperture of *Aristolochia contorta* is porate^27^ or inaperturate^28^. Our results strongly support the observation of Nakonechnaya and Kalachev^28^ because we did not find apertures on the surface of the studied *Aristolochia* pollen grains. Pollen is usually described as inaperturate or 3-zonocolpate within the *Asarum* literature^42–44^. However, our description of the *Asarum* corresponds to the combination of *Asarum europaeum* type^45^ and some Chinese *Asarum*^22^. Moreover, in the case of the *Houttuynia*, we only observed monosulcate grains, similar to a study by Smith and Stockey^26^, although both monosulcate and trichotomosulcate pollen grains were observed^23^.

The exine surface morphology of pollen grains in the studied taxa was similar to that in other Piperales species^21–24,26–29^. The observed variation of exine ornamentation also proved to be a helpful diagnostic characteristic at the inter-/intra generic level. The *Aristolochia* pollen is fossulate without any gemmae/granula, whereas Saururaceae species do not possess a murus pattern. The *Piper* pollen is unique, with microechinate elements without any murus patterns and perforation among studied taxa. The *Asarum* taxa are distinct in two groups based on the gemmae surface patterns (Type II-1 smooth-surfaced vs Type II-2 striate-surfaced). Thus, the presence or absence of gemmae/granula, muri, lumen/perforation on their surface can aid in recognizing certain groups of Korean Piperales.

In the case of the *Asarum mandshuricum* complex, its taxonomic identity remains controversial. *A. mandshuricum* was recognized as two infra-species, *A. mandshuricum* var. *mandshuricum* and *A. mandshuricum* var. *seoulense* based on the presence or absence of trichomes on the petiole^46,47^. However, several studies treated the infra-species, *seoulense*, as synonymous with *A. mandshuricum*^35,37^. A recent taxonomic study revealed that the *A. mandshuricum* complex mostly shares common leaf micromorphological characters^38^, which conflicts with our palynological results. In the present study, *Asarum mandshuricum* complex was distinct in two types (Type II-1 var. *mandshuricum* vs Type II-2 var. *seoulense*). Further in-depth integrative taxonomic studies, including molecular phylogeny, micromorphology, and cytology using abundant samples of *A. mandshuricum* complex, are required to delimitate the taxa to facilitate their identification and classification.

Although orbicule characteristics have been studied in various taxonomic groups, the presence of orbicules and their morphological diversity in Piperales has received little attention^15,16^. Orbicules of selected taxa from Piperales in this study, except for *Houttuynia cordata*, were observed for the first time since the study by Verstraete et al.^16^. The orbicules were identified in all studied taxa, similar to previous research on Piperales^30–32^. The presence of orbicules is regarded as a plesiomorphic feature, common in the early-diverging clades with a trend towards orbicule absence in late-branching^9,16^. Moreover, according to a recent review, the orbicules are also observed in the clade, related to the Piperales^48^. Thus, the presence of orbicules could be considered as a possible symplesiomorphic character of Piperales. A further study focusing on the distribution of orbicules in Magnoliids is required to increase the resolution of their evolutionary trends.

The observed orbicule surface ornamentation was similar to the pollen exine patterns with elements such as muri, gemmae, or granula (Table 2). Orbicule size, in addition to the surface pattern, was significantly correlated with quantitative exine ornamentation elements such as the diameter of gemmae/granula and lumen/perforation, muri branching length, and muri width (Fig. 6). This resemblance between orbicules and pollen surface ornamentations was reported in several taxa^9,12–14,16^. Thus, our results offer additional evidence which implies that a similarly patterned biosynthesis of sporopollenin is possible on a pro-orbicule, as well as on a microspore^16^.

Our dendrogram based on cladistic and phenetic analyses, as well as palynological characters is consistent with the previously reported molecular phylogeny, including the monophyletic lineage of both Piperaceae and Saururaceae^49^. Further studies that include various morphological/micromorphological characteristics are required to understand the evolution of the order Piperales better.

## Conclusion

Inaperturate, monosulcate, tri to pentaporate and very small to large-sized pollen grains, and constantly existing orbicules are consistent characteristics for Korean Piperales. The four types of exine ornamentation and stable orbicule traits may be of great systematic importance for Piperales. Our results strongly support the developing pollen exine hypothesis, whereby orbicules and pollen surface ornamentations are the result of a similar biosynthesis. Further, exine ornamentation patterns may be useful for defining systematic groups at the intra- or interspecific level in *Asarum* as they had a great diversity of palynological features. Palynological characteristics, including orbicules, appear to help reconstruct systematic relationships. The present study helps to understand the diversity in Piperales orbicule morphology, and provides information on pollen and orbicules characteristics. Further studies involving more Piperales taxa, including extensive sampling, will contribute to understanding the evolutionary tendencies of pollen and orbicule characteristics.

## Materials and methods

### Taxon sampling and identification

Mature flowers of 18 accessions (12 species, five genera) were collected from natural habitats to observe the pollen and orbicule morphological features of Korean Piperales (Fig. 1).

All plants described in this paper were collected and used in accordance with the relevant guidelines and regulations. The investigated taxa were neither endangered nor protected. Moreover, all samples were collected with a permit issued from the Korea National Park Service, and voucher specimens were deposited in the Korean Herbarium of Standard Herbal Resources (KIOM) at the Korea Institute of Oriental Medicine, Naju, Korea (see Supplementary Table S1).

Most of the samples were collected from living plants and preserved in FAA solution (40% formalin: 40% glacial acetic acid: 70% ethyl alcohol). To confirm the consistency of palynological characteristics, we compared a minimum of two accessions for each taxon when available.

### Microscopic observation

Prior to observing pollen morphology, all dried floral samples were first examined using a stereomicroscope (SM, Olympus SZX16, Olympus, Tokyo, Japan) to select fully mature anthers. The fully matured anthers were prepared using the critical point drying method (CPD; Moon et al.^15,39^; Song et al.^2,3,14^) for SEM. Dried anthers were rehydrated overnight in a wetting agent Agepon® (Agepon: distilled water, 1:200) (Agfa Gevaert, Leverkusen, Germany). The rehydrated materials were then dehydrated through an ethanol series (50%, 70%, 90%, 95%, and 100% ethanol) at room temperature for 1 h per ethanol concentration. The dehydrated materials were immersed in liquid carbon dioxide (CO_2_) for CPD (SPI-13200JE-AB, SPI Supplies, West Chester, USA). The dried materials were then mounted on aluminum stubs with a double-sided adhesive conductive carbon disk (05073-BA, SPI Supplies, West Chester, USA), and the stubs were coated with platinum using an ion-sputtering device (208HR; Cressington Scientific Instruments Ltd., Watford, United Kingdom) for 90 s. Thereafter, the samples were examined using a field emission scanning electron microscope (FE-SEM, S-4700, Hitachi, Tokyo, Japan) at an accelerating voltage of 5–10 kV and an 8–10 mm working distance.

### Data analysis

The obtained quantitative characteristics were determined using the Digimizer software (Digimizer version 5.4.3, MedCalc Software, Mariakerke, Belgium). Pearson’s correlation coefficients were used to estimate relationships among the following quantitative variables: Orbicule diameter, gemmae/granula diameter, lumen/perforation diameter, muri branching length, and muri width. This statistical analysis was conducted using R, version 3.6.3^50^.

For phenetic analysis, 13 pollen and orbicule characteristics (seven qualitative and six quantitative) and their codes were used (Table 3). Cluster analyses were performed calculating UPGMA using NTSYS-PC 2.1 software^51^ to visualize the relationship between the different species based on seed characters.

**Table 3:** Character list, along with their character states and the codes used in phenetic analysis.

1. Pollen surface ornamentation (SO): gemmate, microreticulate (0), microechinate (1), granulate, microperforate (2), fossulate, perforate (3)
2. Muri width (MW): absence (0), 0.080–0.320 μm (1), 0.321–0.560 μm (2), 0.561–0.800 μm (3)
3. Muri branching length (ML): absence (0), 0.230–0.620 μm (1), 0.621–1.010 μm (2), 1.011–1.400 μm (3)
4. Lumen/perforation diameter (LD): 0.060–0.280 μm (0), 0.281–0.500 μm (1), 0.501–0.720 μm (2), 0.721–0.940 μm (3)
5. Lumen/perforation area (LA): 0.0020–0.1140 μm^2^ (0), 0.1141–0.2260 μm^2^ (1), 0.2261–0.3380 μm^2^ (2), 0.3381–0.4500 μm^2^ (3)
6. Gemmae/granula surface (GS): absence (0), smooth (1), striate (2), microechinate (3)
7. Gemmae/granula diameter (GD): absence (0), 0.100–0.950 μm (1), 0.951–1.800 μm (2), 1.801–2.650 μm (3)
8. Orbicule density (OS): Scattered (0), abundant (1), very abundant (2)
9. Orbicule diameter (OD): 0.220–0.860 μm (0), 0.861–1.502 μm (1), 1.503–2.142 μm (2), 2.143–2.790 μm (3)
10. Orbicule shape (OP): spherical (0), dought-shaped, spherical (1), walnut-shaped, spherical (2), polygonal prism (3)
11. Orbicule surface Ornamentation (OO): psilate (0), rugulate (1), striate (2), microspine (3)
12. Orbicule association (OA): embedded only (0), aggragated, embedded (1), connected via threads (2)
13. Orbicule correlation (SD): muri (0), gemmae (1), granula (2)

The pollen and orbicule terminology was used according to Erdtman^1^, Punt et al.^52^, Hesse et al.^53^, Halbritter et al.^54^, and Verstraete et al.^16^.

## Supplementary Information

Supplementary Table S1.

## Data Availability

All data are fully available without restriction. Moreover, all voucher specimen information of this study is included in this published article (and its “Supplementary Information” files). The plant images and pollen grain, orbicule micro-images can be made available upon requests addressed to J.-H.S.
