# Supplementary material for: Systematic palynology in Korean Piperales with special focus on its exine surface ornamentation and orbicule morphology
Source: Sci Rep. 2022 Mar 9;12:4142. doi: 10.1038/s41598-022-08105-3 (PMC8907175; doi:10.1038/s41598-022-08105-3)
Supplement: Supplementary file 1 — Supplementary Table S1. [file 41598_2022_8105_MOESM1_ESM.doc]

**Table S1.** Voucher specimens of Korean Piperales that are examined in the present study.

| **Taxa** | **Voucher specimens (herbarium acronym)** |
| --- | --- |
| **Aristolochiaceae Juss.** |  |
| ***Aristolochia* L.** |  |
| *A. contorta* Bunge | - Yeongchun-myeon, Danyang-gun, Chungcheongbuk-do (37°02'43.3"N 128°36'21.4"E), B.C. Moon & S. Yang, 28. July 2016. KIOM201701018780 (KIOM) |
| *A. manshuriensis* Kom. | - Banwol-dong, Deokjin-gu, Jeonju-si, Jeollabuk-do, Y.S. Ju & H.J. Kim, 26. April 2009. KIOM200901002588 (KIOM)  - Hwaaksan Mt., Gapyeong-gun, Gyeonggi-do, Y. Ji & B.C. Moon, 25. June 2013. KIOM201201004571 (KIOM) |
| ***Asarum* L.** |  |
| *A. koreanum* J.G.Kim & C.S. Yook ex B.U. Oh | - Geumsusan Mt., Sang-ri, Jeokseong-myeon, Danyang-gun, Chungcheongbuk-do (36°59'09.6"N 128°15'36.4"E), J.-H. Song & S. Yang, 26. April 2018. KIOM201901022336 (KIOM) |
| *A. mandshuricum* (Maxim.) M. Kim & S. So  [≡ *A. heterotropoides* F. Schmidt var. *mandshuricum* (Maxim.) Kitag. ] | - Hwayasan Mt., Cheongpyeong-myeon, Gapyeong-gun, Gyeonggi-do (37°41'36.4"N 127°24'13.9"E), J.-H. Song & S. Yang, 27. April 2018. KIOM201901022356 (KIOM)  - Cheonmasan Mt., Hopyeong-dong, Namyangju-si, Gyeonggi-do (37°40'28.8"N 127°15'25.3"E), J.-H. Song & S. Yang, 28. April 2018. KIOM201901022351 (KIOM) |
| *A. mandshuricum* var. *seoulense* (Nakai) M. Kim &  S. So [=*A. mandshuricum*] | - Anchang-ri, Jijeong-myeon, Wonju-si, Gangwon-do (37°20'32.6"N 127°48'54.3"E), J.-H. Song & S. Yang, 26. April 2018. KIOM201901022340 (KIOM)  - Cheonmasan Mt., Onam-eup, Namyangju-si, Gyeonggi-do (37°40'48.2"N 127°15'44.8"E), J.-H. Song & S. Yang, 28. April 2018. KIOM201901022349 (KIOM) |
| *A. misandrum* B.U. Oh & J.G. Kim | - Gyorae-ri, Jocheon-eup, Jeju-si, Jeju-do (33°25'56.1"N 126°42'05.4"E) J.-H. Song & S. Yang, 01. May 2018. KIOM201901022370 (KIOM) |
| *A. patens* (Yamaki) M. Kim & S. So | - Seondosan Mt., Sangdang-gu, Cheongju-si, Chungcheongbuk-do (36°37'50.7"N 127°33'24.7"E), J.-H. Song & S. Yang, 21. April 2018. KIOM201901022344 (KIOM) |
| *A. sieboldii* Miq. | - Wontongsa Temp., Anseong-myeon, Muju-gun, Jeollabuk-do, Y. Ji, 07. May 2013. KIOM201401009283 (KIOM) |
| *A. versicolor* (K. Yamaki) Y.N. Lee | - Hwayasan Mt., Cheongpyeong-myeon, Gapyeong-gun, Gyeonggi-do (37°41'29.1"N 127°24'33.8"E), J.-H. Song & S. Yang, 27. April 2018. KIOM201901022353 (KIOM) |
| **Saururaceae Rich. ex T. Lestib.** |  |
| ***Houttuynia* Thunb.** |  |
| *H. cordata* Thunb. | - Gagok-dong, Miryang-si, Gyeongsangnam-do (35°28'55.9"N 128°45'57.7"E), H.J. Choi, 03. May 2019. MFDS-V-11236 (KIOM)  - Buk-myeon, Ulleung-gun, Gyeongsangbuk-do, Korea, Y. Ji & B.C. Moon, 19. June 2012. KIOM201201004718 (KIOM) |
| ***Saururus* L.** |  |
| *S. chinensis* (Lour.) Baill. | - Yongsu-ri, Hangyeong-myeon, Jeju-si, Jeju (33°19'11.6"N 126°10'49.8"E), J.H. Lee, 12. August 2019. MFDS-V-6846 (KIOM)  - Andong-si, Gyeongsangbuk-do, Korea (36°35'40.6"N 128°32'48.2"E), B.C. Moon, 03. July 2014. KIOM201501013841 (KIOM) |
| **Piperaceae Giseke** |  |
| ***Piper* L.** |  |
| *P. kadsura* (Choisy) Ohwi | - Takakusa-yama Mt., Utsutani, Okabe-cho, Fujieda-shi, Shizuoka, Honshu, F. Konta, 26. May 2009. 35716 (SNU)  - Gamsan-ri, Andeok-myeon, Seogwipo, Jeju-do, South Korea (33°15'32.8"N 126°21'24.3"E), J.-H. Song & S. Yang, 17. June 2020. SJH_KIOM-2021-523 (KIOM) |
